# Fighting COVID-19 Misinformation through an Online Game Based on the Inoculation Theory: Analyzing the Mediating Effects of Perceived Threat and Persuasion Knowledge

**DOI:** 10.3390/ijerph20020980

**Published:** 2023-01-05

**Authors:** Jinjin Ma, Yidi Chen, Huanya Zhu, Yiqun Gan

**Affiliations:** 1 School of Psychological and Cognitive Sciences and Beijing Key Laboratory of Behavior and Mental Health, Peking University, Beijing 100871, China; 2 Department of Psychology, School of Humanities and Social Sciences, Beijing Forestry University, Beijing 100083, China

**Keywords:** COVID-19 misinformation, fake news, inoculation theory, online intervention, randomized controlled trial

## Abstract

The COVID-19 pandemic was accompanied by the rapid spread of misinformation through social media platforms. This study attempted to develop an online fake news game based on the inoculation theory, applicable to the pandemic context, and aimed at enhancing misinformation discrimination. It also tested whether perceived threat and persuasion knowledge serve as underlying mechanisms of the effects of the intervention on misinformation discrimination. In Study 1, we used online priming to examine the influence of inoculation on misinformation discrimination. In Study 2, we developed an online fake-news-game-based intervention and attempted to validate its effectiveness through a randomized controlled trial while also exploring the mediating roles of perceived threat and persuasion knowledge. In Study 1, brief inoculation information priming significantly enhanced the ability to recognize misinformation (*F*(2.502) = 8.321, *p* < 0.001, η_p_^2^ = 0.032). In Study 2, the five-day game-based intervention significantly enhanced the ability to recognize misinformation (*F*(2.322) = 3.301, *p* = 0.038, η_p_^2^ = 0.020). The mediation effect of persuasion knowledge was significant (β = 0.025, *SE* = 0.016, 95% CI = [0.034, 0.075]), while that of perceived threat was not significant. Online interventions based on the inoculation theory are effective in enhancing misinformation discrimination, and one of the underlying mechanisms of this effect lies in its promotion of persuasion knowledge.

## 1. Introduction

With the outbreak of the COVID-19 pandemic, anxiety and panic caused by the uncertainty of the pandemic made the need for information urgent and pressing, which also provided the foundation that many rumormongers needed for spreading a plethora of related misinformation on social media platforms [1]. The spread of COVID-related rumors is dangerous because it can dissuade people from adopting health behaviors that help protect themselves and others [2]. Exposure to misinformation has been found to lead to associated false health beliefs that influence individuals’ intentions and health behaviors [3] in accordance with the theory of planned behavior. In particular, COVID-related misinformation focuses heavily on virus transmission and protection [4], influencing individuals’ compliance with pandemic protection behaviors, such as hand washing, wearing masks, maintaining social distance, and vaccination, and leading to increased morbidity [5]. The widespread spread of misinformation has become a global challenge and burden to individual health, public health, and governments [6]. Accordingly, the World Health Organization (WHO) has indicated that the pandemic triggered another crisis, namely, the “infodemic” [7]. This led the WHO to prioritize combating the infodemic and call for a unified response from governments, companies, and social organizations.

Refuting rumors is a common method used to tackle infodemics, although researchers suggest that once misinformation is ingrained in one’s memory, it is difficult to change [8]. Therefore, improving or developing enhanced methods for debunking misinformation may be insufficient to efficiently tackle it [9], and preemptive or preventive approaches can be more useful for dealing with infodemics. Since misinformation spreads on the Internet in a similar manner to virus replication [10], methodologies based on the inoculation theory have been considered promising solutions and have been receiving increasing attention across academic fields in recent years.

### 1.1. Inoculation Theory

The inoculation theory was originally pioneered by McGuire in the 1960s [11] and aims to guide people against the effects of external persuasion in a similar way to biological immunity. Similar to vaccination, inoculation refers to the exposure of individuals to information containing weakened arguments against the attitudes they hold, thus allowing individuals to prevent future attacks on such attitudes [12]. Inoculation is thought to be divided into two processes: (1) threat, which is the recognition that current perceptions might be challenged, and (2) the active process of presenting opposing arguments to counteract the challenge [12]. Interventions derived from the inoculation theory are already widely used to reinforce general views on topics such as vaccination and other health behaviors [13]. Nonetheless, the traditional inoculation theory has two important limitations. First, it presupposes that individuals’ initial beliefs about an issue are accurate, which tends not to be the rule (or to be hard to achieve) in real life when the topics are controversial and especially so for widely spread, inaccurate rumors. Second, rebuttals are made in a passive rather than an active manner. These two limitations greatly restrict the scalability and generalizability of the “inoculation” metaphor [14].

To address these two limitations of the traditional view, Roozenbeek and Van der Linden [15] proposed a broad-spectrum inoculation theory, focusing on the application of the inoculation metaphor to common strategies for generating misinformation, rather than solely on specific persuasive content. They also developed an intervention based on this expanded theory, specifically a free online game. In the game, players learn the characteristics of six common misinformation types and misinformation generation strategies and then enter a simulated social media environment (Twitter), where they are asked to discriminate the information they receive between real and fake news [16]. They found that the intervention effectively improved rumor discrimination, thereby demonstrating the effectiveness of the broad-spectrum inoculation theory.

Roozenbeek and Van der Linden, after reviewing the extensive literature on rumors, categorized the types of rumors into conspiracy, polarization, impersonation, emotion, discredit, and rolling [15]. Since public events or crises that occur abruptly are characterized by suddenness, complexity, and uncertainty, the related misinformation tends to have characteristics that differ from normal misinformation. For example, Brennen et al. [4] analyzed rumors in the early stages of the COVID-19 outbreak and found that in addition to conspiracy and impersonation rumors, some of them focused on spreading unverified, false knowledge about COVID-19. Therefore, this study proposes an expansion of the broad-spectrum inoculation theory by Roozenbeek and Van der Linden and the original six categories by introducing an additional category, namely, spreading false knowledge. These seven categories were targeted in the intervention of the current study.

### 1.2. Underlying Mechanisms of the Effect of Inoculation Interventions

Although the effectiveness of broad-spectrum inoculation theory for helping people to discriminate rumors in the context of vaccination was initially confirmed, the mechanisms underlying the intervention effects remain unknown. In the traditional inoculation theory, a threat is considered the first step in building antibodies and the motivation to generate defenses [17]. In fact, it has been demonstrated that people can perceive threats with ease after reading inoculation messages [18], leading explicit threat warnings to become typical methods in inoculation interventions. Compton and Ivanov [19] found that the warning component may be the most significant factor in generating threats and that considering the threat could enhance resistance to subsequent misinformation. Accordingly, perceived threat may be a mechanism of the effect of an inoculation intervention on misinformation discrimination.

Persuasion knowledge may also be an important mechanism of this effect. Persuasion knowledge refers to how individuals respond to persuasion attempts and their beliefs about the tactics of how messages such as advertising can persuade them [20]. Conceptually speaking, believing in rumors is the result of persuasion processes, and the inoculation theory is one of the six foundational components of the persuasion theory [21]. Furthermore, the inoculation theory has been applied as an implicit theoretical basis for coping responses in models of persuasion knowledge [22]. However, limited research has explored the role of persuasion knowledge in inoculation interventions. Persuasion knowledge has been shown to be an important resource protecting people from being influenced by misinformation [23], in that higher levels of persuasion knowledge can urge individuals to engage in a stronger rebuttal of misinformation and thus protect against it [22]. In summary, persuasive knowledge is closely related to the inoculation theory and warrants further, in-depth research.

### 1.3. Present Research

Against this background, a question emerges: considering the massive spread and impact of misinformation during the COVID-19 pandemic, could broad-spectrum inoculation-based interventions be effective in enhancing the public’s misinformation discrimination? If yes, what are the mechanisms by which the interventions take effect?

Based on these guiding questions, this study set out to develop an online fake news game based on the inoculation theory, applicable to the COVID-19 pandemic context, and to test whether perceived threat and persuasion knowledge can serve as underlying mechanisms of the effect of inoculation-based interventions on misinformation discrimination. In misinformation intervention studies, the ability to recognize misinformation is a common indicator of measurement. It refers to the ability to discriminate between facts and misinformation [16]. Considering that misinformation propagation often achieves viral growth through reposting on online platforms [10], persuasive intent behavior is a more ecologically valid indicator. This refers to behaviors such as retweets and likes triggered by misinformation, as measured by the willingness to retweet misinformation [24]. These two indicators were used in this study to measure misinformation discrimination.

In particular, in Study 1, we used online priming to examine the influence of inoculation on misinformation discrimination. In Study 2, we developed an online fake news game to validate the effectiveness of the inoculation intervention through a randomized controlled trial and explored the mediating role of perceived threat and persuasion knowledge in the relation between the inoculation intervention and misinformation discrimination. Based on the literature review presented above, we propose the following hypotheses:

**Hypothesis 1** **(H1).** *The online inoculation intervention effectively enhances misinformation discrimination, including the ability to recognize misinformation and the persuasive intent of misinformation*.

**Hypothesis 2** **(H2).** *Perceived threats and persuasion knowledge significantly mediate the relationship between the online inoculation intervention and misinformation discrimination*.

## 2. Study 1: Influence of Inoculation on Misinformation Discrimination: An Online Priming Study

Study 1 aimed to test the effect of short-term, online inoculation priming on the ability to recognize misinformation and the persuasive intent of misinformation. The study was conducted amid the COVID-19 outbreak in China.

### 2.1. Methods

#### 2.1.1. Participants

Healthy participants aged 18–55 years were recruited through convenience sampling from WeChat, a Chinese social media platform. Three hundred fifty-eight participants enrolled in this study by completing the recruitment questionnaire in June 2020. An initial understanding of virus characteristics had been gained during this period, the epidemic had been steadily controlled in China, and the prevention and control work had been transformed from an emergency state to a regular prevention state [25]. Two attention check items were included in the questionnaire (“*I have never used mobile phones*”; “*Select very strongly*”), and participants who failed in either were excluded from the study. After applying the exclusion criteria, 353 valid participants remained in the pretest, 325 in the posttest, and 280 in the follow-up period. The final data set contained the responses of 256 participants (*M* = 25.46, *SD* = 7.30; 69 men), providing valid questionnaires across all time points. The sample size was calculated using G-power 3.1; given a medium effect size of 0.25 and a significance level of 0.05, 158 participants were needed to achieve a statistical power of 80%. The final sample size of this study satisfied the requirements for statistical power.

The participants signed the online consent form prior to study onset. This study was approved by the Peking University Committee for the Protection of Human and Animal Subjects.

#### 2.1.2. Materials

- Demographic Characteristics

Basic demographic information of participants was collected, including sex, age, occupation, and subjective socioeconomic status (SES). Subjective SES was measured using the MacArthur scale, which asked the participants to place themselves on a ladder scale from 1 (bottom of the ladder; people who are the worst off) to 10 (top of the ladder; people who are the best off) relative to their societal communities in terms of education, income, and occupation [26].

- Ability to Recognize Misinformation

Participants’ ability to recognize misinformation was measured using three parallel tests across the three time points (pretest, posttest, and follow-up). This was implemented to avoid the repetitive testing of items that participants could search for online after each test.

**Item Pool and Item Selection.** The initial pool of items for testing the ability to recognize misinformation was generated from the China Internet Joint Disinformation Platform (sponsored by the Central Network Information Office Illegal and Undesirable Information Reporting Center), the WeChat Disinformation Platform (sponsored by Tencent Computer System Co., Ltd. (Shenzhen, China)), and the Ding Xiang Doctor Disinformation Platform. The initial item pool contained 100 pieces of fake news (e.g., “*Being diagnosed with COVID-19 during pregnancy results in a child with other health problems*”) and 100 pieces of real news (“*Most symptomatic COVID-19 cases are mild*”). To construct three different sets of parallel questionnaires to be used across time points, 30 pieces of news each for fake and real news were selected randomly from the item pool. These selected items were then presented to 153 participants, who were asked to rate the reliability of the fake news and real news on a scale from 1 (*unreliable*) to 7 (*reliable*) [16]. These participants did not overlap with Study 1 participants and were also recruited through WeChat. They indicated their consent to participate by signing an informed consent form and then completed the news ratings through an online questionnaire. Nine participants who completed the test in less than 180 s were excluded, resulting in a final sample of 144 participants aged 18–55 years (*M* = 37.88, *SD* = 7.97; 50 men).

**Item Selection and Randomization.** Item difficulty analysis was conducted to test whether the items were reasonable. For each item, a score between 1 and 3 was considered the lower region and a score between 5 and 7 was considered the upper region. An item was excluded if the percentage of its score in the upper or lower region was over 90%.

The item discrimination index was also used to measure how well the test items were able to distinguish between participants. Participants who scored over 295 (*n* = 37; 27% of the total sample) were coded as the high-score group, and participants with scores lower than 261 (*n* = 37; 27% of the total) were assigned to the low-score group. An independent-group t-test was conducted to compare the low and high groups for each item. Items that did not distinguish between groups were excluded from the item pool.

The remaining items included 15 facts and 24 pieces of misinformation. To ensure that the parallel tests were as homogeneous as possible, the items were divided into five equal groups according to the score (the third group had four pieces of misinformation), which means that items with close scores were grouped together. Three items were randomly selected within each group to be used in the three parallel tests. One-way analysis of variance (ANOVA) revealed no significant differences in the items between the three parallel tests (*F*(2,286) = 1.171, *p* = 0.171, η_p_^2^ = 0.012).

**Items in Parallel Tests.** Each of the three parallel tests contained five pieces of misinformation and five facts. During the test, the participants were asked to rate these 10 pieces of news from 1 (*unreliable*) to 7 (*reliable*). Items containing misinformation and fake news were reverse-scored. This means that individuals with higher scores for the ability to recognize misinformation are more reliable with facts and less reliable with misinformation.

- Persuasive Intent of Misinformation

We measured the persuasive intent of misinformation, which is one’s behavioral change after being exposed to misinformation, using a scale adapted from the selling and persuasive intent scale developed by Wojdynski and Evans [24]. It contains three parallel tests, each of which was presented after the tests measuring the ability to recognize misinformation. Each parallel test simulates a real social media platform and evaluates the willingness to repost five pieces of misinformation and five facts. The tests were rated on a 7-point Likert scale ranging from 1 (*strongly unwilling*) to 7 (*strongly willing*). The scoring for misinformation is reverse-scored, with higher scores indicating less intention to repost the information due to misinformation persuasion. The reliability coefficients for the items measuring the persuasive intent of facts were 0.756, 0.812, and 0.866 in the pretest, posttest, and follow-up period, respectively; those for the items measuring the persuasive intent of misinformation were 0.806, 0.770, and 0.826, respectively.

#### 2.1.3. Priming

**Coding Materials.** The materials for coding were selected from a total of 85 pieces of news reports in the item pool, excluding the 15 pieces of fake news used to measure the ability to recognize misinformation.

**Coding.** The strategies used for producing misinformation were categorized into seven types: conspiracy, polarization, impersonation, emotion, discredit, trolling [4,27], and the one proposed for this study, spreading false knowledge. Two graduate students majoring in psychology independently coded each fake news item into one of these seven common strategies before comparing and discussing the differences in their coding. When they could not reach an agreement on an item, that item was excluded.

**Coding Adjustments.** Based on careful discussions between the two graduate students, a solution with four strategy types was reached: conspiracy, impersonation, emotion, and spreading false knowledge.

**Recoding.** After determining this solution, the two students recoded and discussed the data, and the initial coding consistency was 83%.

**Priming Material.** Eight pieces of misinformation and four facts were randomly selected from the item pool. During priming, participants were first presented with news reports and asked to determine whether they were facts or misinformation. Feedback on the responses, misinformation characteristics (only for misinformation), and further explanations were presented immediately afterwards. For example, one piece of misinformation presented to participants was “*Being diagnosed with COVID-19 during pregnancy results in a child with other health problems*.” An incorrect answer prompted responses similar to this one:

“*Wrong. This statement conveys misinformation. It strategically spreads false knowledge of the characteristics of the virus. The National Health Council notes that there is no evidence of mother-to-child transmission of the new coronavirus. If necessary, you can discuss with your obstetrician about whether to get pregnant*.”

#### 2.1.4. Procedure

The participants completed the questionnaires via the Surveystar platform in a neutral and calm environment. During the pretest (Day 1), participants first completed a survey on their demographic information and then completed various self-reported questionnaires to measure their ability to recognize misinformation and the persuasive intent of misinformation.

In the posttest (Day 2), based on the last digits of their phone numbers, the participants were allocated to the priming group (odd digits) or the control group (even digits). Immediately after priming (priming group) or no activity (control group), all participants completed the same self-reported measures present in the pretest again. In the follow-up (after one week), the participants were asked to complete the same battery of tests once more.

#### 2.1.5. Statistical Analysis

All analyses were conducted using SPSS version 26.0. A mixed-measures ANOVA was used to test the differences in the ability to recognize misinformation and the persuasive intent of misinformation between the control and priming groups across time points.

### 2.2. Results

#### 2.2.1. Baseline Analysis

One-way ANOVA was conducted to test whether there was a significant difference between the priming and control groups at baseline. No significant differences were found in the ability to recognize misinformation (*F*(1,254) = 0.267, *p* = 0.606, η_p_^2^ = 0.001), nor in the persuasive intent of misinformation (*F*(1,254) = 0.323, *p* = 0.570, η_p_^2^ = 0.001). The descriptive statistics are shown in Table 1.

#### 2.2.2. Effect of Inoculation Priming on Misinformation Discrimination

**Figure 1:**
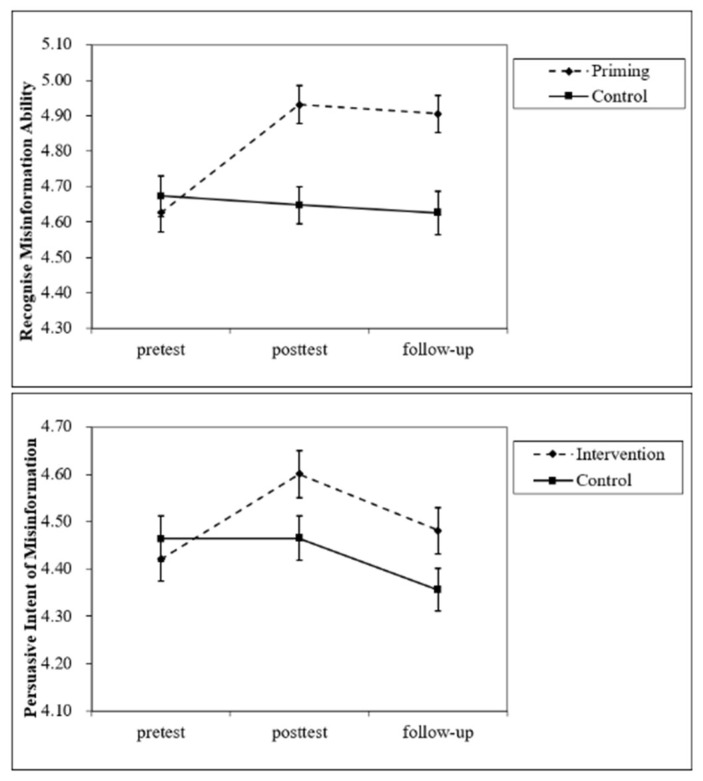
Interaction effect between group and time on misinformation discrimination in Study 1.

To test whether inoculation priming could improve the ability to recognize misinformation (H1), a mixed-design ANOVA was conducted with the group (priming vs. control) and time (pretest vs. posttest vs. follow-up) as independent variables; the ability to recognize misinformation as the dependent variable; and sex, age, and SES as covariates. A significant interaction effect of the priming group and time was found (*F*(2.502) = 8.321, *p* < 0.001, η_p_^2^ = 0.032). A simple effect test demonstrated that the priming group showed a higher ability to recognize misinformation in the posttest (95% confidence interval (CI) = [−0.495, −0.151]) and follow-up (95% CI = [−0.446, −0.111]), whereas the control group showed no changes (Figure 1). Moreover, the main effect of the priming group was significant (*F*(1,251) = 9.072, *p* = 0.003, η_p_^2^ = 0.035), in that the ability to recognize misinformation was significantly higher in the priming group than in the control group. This result is consistent with H1.

To test whether inoculation priming could change how people dealt with the persuasive intent of misinformation (H1), the same mixed-design ANOVA was conducted with the persuasive intent of misinformation as the dependent variable. A significant interaction effect between the priming group and time was found (*F*(2,502) = 3.266, *p* = 0.042, η_p_^2^ = 0.013). A simple effect test demonstrated that the priming group showed higher scores for the persuasive intent of misinformation in the posttest (95% CI = [−0.320, −0.041]), but not at follow-up. For the control group, there were no changes (Figure 1). No significant main effect was found. This result is consistent with H1.

### 2.3. Discussion

Study 1 longitudinally explored the effectiveness of an inoculation intervention on misinformation discrimination during the COVID-19 pandemic. First, we confirmed that there were no significant differences between groups at baseline; then, our results showed a significant improvement in the ability to recognize misinformation in the priming group compared to the control group after completing the priming, and this positive effect persisted for one week (follow-up). The findings for the persuasive intent of misinformation also showed a significant improvement in the priming group after priming, whereas no significant change was observed in the control group; this remained consistent at follow-up.

Based on these results, Study 2 was conducted using a self-serve online fake news game, which may be a more applicable intervention approach under the constraints imposed by the social distancing measures related to the COVID-19 pandemic. It served to verify the effectiveness of online inoculation interventions through a randomized controlled trial and explore the mediating roles of perceived threat and persuasion knowledge.

## 3. Study 2: Influence of an Online Inoculation Intervention on Misinformation Discrimination: A Randomized Controlled Trial

Study 2 aimed to test the effect of the online inoculation intervention on the ability to recognize misinformation and the persuasive intent of misinformation. The study was conducted with a randomized controlled trial.

### 3.1. Methods

#### 3.1.1. Participants

The prior sample size calculation using G-power 3.1 showed the need for at least 158 participants. Considering the potential for dropouts, we recruited 280 participants with convenience sampling. The inclusion criteria were residing in China, aged between 18 and 55 years, had not participated in Study 1, and capable of operating smartphones and WeChat apps. Three hundred participants enrolled in this study by completing the recruitment questionnaire, which occurred in early July 2020 when the pandemic was still in its regular prevention and control phase. The final sample comprised 167 participants (31 men), with an average age of 22.99 years (*SD* = 6.30). This study was approved by the Peking University Committee for Protecting Human and Animal Subjects, and all participants provided written informed consent prior to the experiment.

#### 3.1.2. Procedure

The participants were randomized into either the intervention or control group using a sample randomization program called FFcell. After allocation, participants were added by the researcher to two separate WeChat group chats corresponding to whether they were in the intervention or control group. Participants were unaware of the other group chat, and they were blinded to the experiment.

**Figure 2:**
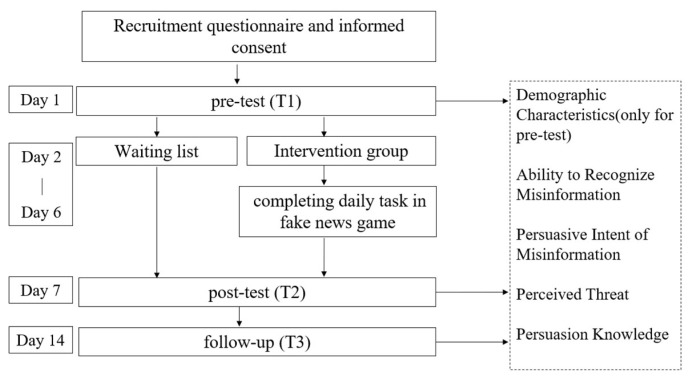
Procedure for Study 2.

On Day 1 (T1), all participants responded to a questionnaire including items on demographic characteristics, the ability to recognize misinformation, the persuasive intent of misinformation, perceived threat, and persuasion knowledge of online misinformation on the Surveystar platform. Then, starting on Day 2 and lasting for five consecutive days, participants in the intervention group were required to complete daily intervention tasks in a WeChat Mini program, which was posted in the intervention group chat. At the end of the intervention (Day 7, T2), participants in both the intervention and control groups completed posttest measures. The follow-up occurred one week later (T3). The posttest and follow-up measures were the same as in the pretest, except that no demographic variables were included (Figure 2). This study was pre-registered on Open Science Framework on 9 July 2020 (osf.io/642ut (accessed on 16 June 2022)).

#### 3.1.3. Intervention Procedure

In this study, the intervention was administered via the WeChat Mini program in the form of an online game. The interventions in the WeChat application were developed based on the intervention materials from Study 1; specifically, participants were presented with numerous pieces of news and decided whether the news was a fact or misinformation. Participants then received feedback on their answers and were provided with explanations if the news contained misinformation. The intervention lasted for five consecutive days, and 12 different pieces of news materials were presented randomly each day. To ensure that participants were seriously engaged in the intervention, we excluded those who responded correctly on less than 50% of the items.

To gamify the intervention and increase adherence, we added competition and achievement elements. The competition system recorded the scores of participants regarding their responses to the news, and all participants’ accumulated scores were ranked. The scores were influenced by whether the participant assessed the news correctly and the speed of their response. The achievement system provided badges when the cumulative percentage of correct answers to questions of a specific misinformation type (i.e., conspiracy, impersonation, emotion, and spreading false knowledge) reached 80%. Participants were informed of all elements in the Mini program in the form of a prompt box the first time they participated in the game.

#### 3.1.4. Materials

- Demographic Characteristics

Participants’ basic demographic information data were collected, including sex, age, occupation, and subjective SES.

- Ability to Recognize Misinformation

The tests used in Study 2 to measure the ability to recognize misinformation were the same as those for Study 1.

- Persuasive Intent of Misinformation

The scale for measuring the persuasive intent of misinformation in Study 2 was the same as that used in Study 1. The reliability coefficients for the items measuring the persuasive intent of facts were 0.809, 0.767, and 0.813 in the pretest, posttest, and follow-up, respectively; those for the items measuring the persuasive intent of misinformation were 0.814, 0.788, and 0.839, respectively.

- Perceived Threat Toward COVID-19-Related News

Perceived threat was measured using a standard questionnaire from a study regarding the inoculation theory [19]. It contains six opposing adjectives: threatening/non-threatening, harmful/non-harmful, dangerous/non-dangerous, risky/non-risky, calm/anxious, and scary/non-scary, which were rated on a 7-point Likert scale ranging from 1 (*strongly disagree*) to 7 (*strongly agree*). The adjectives were used to answer the following question: “*How do you feel about news regarding future pandemics you may encounter?*” The reliability coefficients of the scale were 0.908, 0.948, and 0.957 in the pretest, posttest, and follow-up, respectively.

- Persuasion Knowledge of Online Misinformation

Persuasion knowledge was measured using a scale adapted from the Pricing Persuasion Knowledge Scale [28]. It contained three items evaluating one’s knowledge of online misinformation persuasion tactics (“*I understand that Internet rumormongers can use specific strategies to attract attention*”). It was rated on a 7-point Likert scale ranging from 1 (*strongly disagree*) to 7 (*strongly agree*). The reliability coefficients of this scale were 0.748, 0.822, and 0.821 in the pretest, posttest, and follow-up, respectively.

#### 3.1.5. Statistical Analysis

All analyses were conducted using SPSS version 26.0 and PROCESS version 3.5. A mixed-measures ANOVA was used to test the differences in the ability to recognize misinformation and the persuasive intent of misinformation between the control and intervention groups across all time points. Parallel mediation models were used to test the mediating effect of persuasion knowledge (posttest) and perceived threat (posttest) on the relationship between groups and the ability to recognize misinformation (posttest).

### 3.2. Results

#### 3.2.1. Attrition Analysis

**Figure 3:**
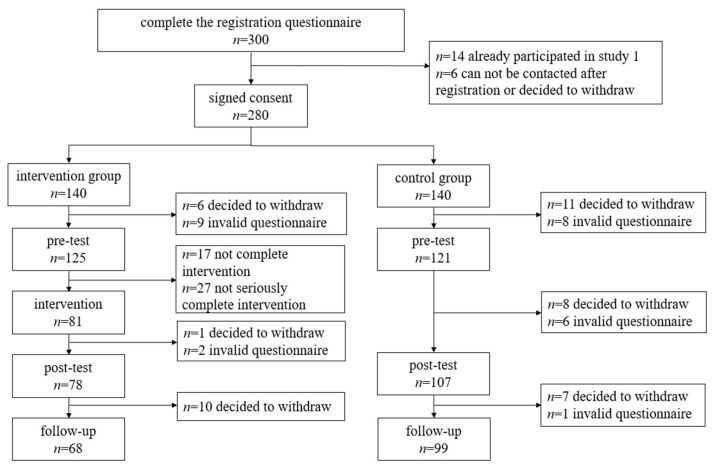
CONSORT flow diagram.

In total, 280 participants met the requirements of the study, and 167 completed the study (Figure 3). To avoid non-random attrition, differences between attrition and valid participants were analyzed for the main variables and demographic variables at the pretest. No significant differences were found in sex (*χ*^2^ = 0.032, *p* = 0.857); age (*F*(1,247) = 0.664, *p* = 0416, η_p_^2^ = 0.003); the ability to recognize misinformation; or the persuasive intent of misinformation (Pillai’s trace = 0.022, *F*(2,246) = 2.786, *p* = 0.064, η_p_^2^ = 0.022).

#### 3.2.2. Baseline Analysis

One-way ANOVA was conducted to test whether there was a significant difference between the intervention and control groups at baseline. No significant difference was found in the ability to recognize misinformation (*F*(1,165) = 0.002, *p* = 0.964, η_p_^2^ < 0.001) or the persuasive intent of misinformation (*F*(1,165) = 0.417, *p* = 0.520, η_p_^2^ = 0.003) at baseline. The descriptive statistics are shown in Table 2.

#### 3.2.3. Effect of the Inoculation Intervention on Misinformation Discrimination

**Figure 4:**
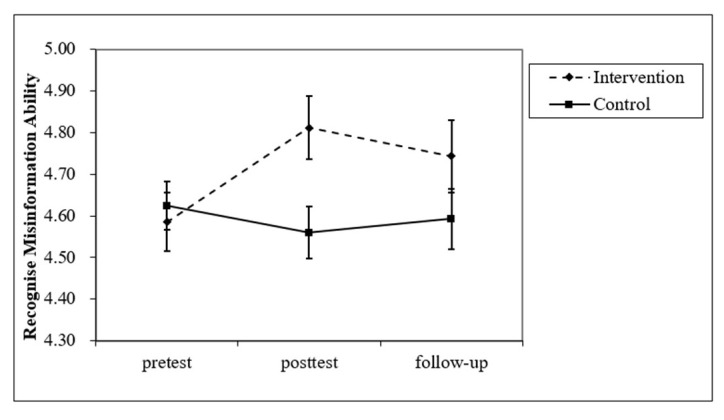
Interaction effect between group and time on misinformation discrimination in Study 2.

To test whether inoculation priming could improve the ability to recognize misinformation (H1), a mixed-design ANOVA was conducted with the group (intervention vs. control) and time (pretest vs. posttest vs. follow-up) as independent variables; the ability to recognize misinformation as the dependent variable; and sex, age, and SES as covariates. A significant interaction effect between the intervention group and time was found (*F*(2,322) = 3.301, *p* = 0.038, η_p_^2^ = 0.020). A simple effect test showed that for the intervention group, the ability to recognize misinformation in the posttest was significantly higher than in the pretest (95% CI = [−0.438, −0.018]), but there was no significant difference between the follow-up, pretest, and posttest periods; for the control group, there was no significant difference between the pretest, posttest, and follow-up periods (Figure 4). No significant main effects were observed. This result is consistent with H1, but the effect was not maintained by the time of follow-up.

To test whether the intervention could change the persuasive intent of misinformation (H1), the same ANOVA was conducted with the persuasive intent of misinformation as the dependent variable. The results showed a significant main effect of the intervention (*F*(1,161) = 5.711, *p* = 0.018, η_p_^2^ = 0.034), with a higher persuasive intent of misinformation in the intervention group than in the control group. H1 was not verified for the persuasive intent of misinformation.

#### 3.2.4. Mediating Effects of Persuasion Knowledge and Perceived Threat

**Figure 5:**
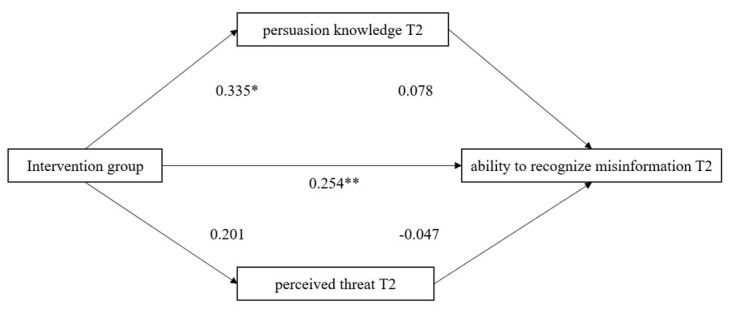
Mediation effect of persuasive knowledge and perceived threat. ** *p* < 0.01; * *p* < 0.05.

A mediation model (model 4 in PROCESS) was constructed with the group (intervention vs. control) as the independent variable, the ability to recognize misinformation (posttest) as the dependent variable, and persuasion knowledge (posttest) and perceived threat (posttest) as the mediation variables. We also controlled for the effects of sex, age, SES, the ability to recognize misinformation (pretest), persuasion knowledge (pretest), and perceived threat (pretest). The results indicated that the mediation effect of persuasion knowledge holds (*β* = 0.025, *SE* = 0.016, 95% CI = [0.034, 0.075]), while that of perceived threat does not hold (*β* = −0.009, *SE* = 0.014, 95% CI = [−0.057, 0.0062]) (Figure 5). H2 was verified for persuasion knowledge, while the hypothesis was not verified for perceived threat.

### 3.3. Discussion

Study 2 developed a self-help misinformation game to verify the effectiveness of the online inoculation intervention on the ability to recognize misinformation and the persuasive intent of misinformation through a randomized controlled trial. It also explored the mediating effect of perceived threat and persuasion knowledge. The results showed that after completing the five-day online intervention, the intervention group demonstrated a significant improvement in the ability to recognize misinformation compared with the control group, although it did not persist in the follow-up period. The persuasive intent of misinformation was also significantly higher in the intervention group than in the control group. In addition, the results of this study showed that in the intervention group, persuasion knowledge significantly mediated the relationship between the inoculation intervention and misinformation discrimination. Notwithstanding, the mediating effect of perceived threat was not significant, providing evidence to explain the mechanism of the online inoculation intervention for improving misinformation discrimination.

## 4. General Discussion

This study developed an online fake news game based on the inoculation theory in the context of the COVID-19 pandemic and examined the mechanisms by which an inoculation intervention takes effect. Study 1 used inoculation information as a brief priming session and found significant improvements in both the ability to recognize misinformation and the persuasive intent of misinformation after priming, which were maintained until seven days after the intervention. This result supports H1. In Study 2, we developed a more complete, five-day online fake news game and examined the effects of the intervention using a randomized controlled trial design. It demonstrated the effectiveness of the intervention in improving the ability to recognize misinformation, but the effects did not persist in the follow-up. Persuasion knowledge served as a mediating mechanism to explain the increase in the ability to recognize misinformation, whereas perceived threat did not; thus, H2 was partially supported.

### 4.1. Effectiveness of the Inoculation Intervention

In both Studies 1 and 2, broad-spectrum inoculation was found to be effective in enhancing the ability to recognize misinformation, consistent with prior research on the inoculation theory [16,29]. Study 2, a randomized control trial, provides stronger evidence and further validates the effectiveness of the online inoculation intervention. Researchers interested in topics surrounding misinformation have focused on related preventive measures and interventions in recent years [15,30], and the inoculation theory has been described as a major method to curb the impact of misinformation. The current findings underpin the equal applicability of inoculation interventions for dealing with misinformation regarding pandemic scenarios and for tackling infodemics.

The evaluation index of the intervention’s effectiveness, the ability to recognize misinformation, and the persuasive intent of misinformation measured the reliability and reposting intention of both factual news and misinformation. Based on this, we inferred that the intervention did not increase the level of unreliable reposting for all types of information but rather enhanced the reliability with facts and reduced reliability with misinformation. To further clarify the intervention efficacy target, future research could attempt to dichotomize yes and no judgments for materials containing misinformation and facts and use the correctness of the judgments as a test for the intervention efficacy.

However, the duration of the effects of the intervention differed between Studies 1 and 2. In Study 1, the effect was maintained for a longer period (vs. Study 2), despite it comprising only a brief priming session; in Study 2, the effect lasted for a shorter period, and this was despite the longer, five-day intervention compared with that in Study 1. This may be related to the process of mastering knowledge, which the saying “The more you learn, the harder it gets” efficiently describes. That is, although the period for learning the knowledge of misinformation in Study 2 was longer than that in Study 1, the relatively deeper learning process in Study 2 is also more likely to have caused confusion among participants.

On the one hand, researchers have shown that the repeated activation of memory makes it more likely for people to produce false memories and requires sufficient reconsolidation to ensure correct recall [31]. This means that multiple sessions of the fake news judgment task in our game may have led people to become confused about their memories of the knowledge on misinformation, and that this knowledge may only be truly mastered when enough practice is allowed, as this enables people to form a stable, long-term memory of the topic. On the other hand, knowledge mastery and self-evaluation are closely related, in that self-evaluation affects individuals’ performance on tasks [32]. As the Dunning–Kruger effect suggests, self-evaluation decreases at first, but it then increases as knowledge is mastered; then, after a learning period, individuals become more likely to realize the limitations of their abilities and to develop negative emotions (e.g., frustration and self-doubt) [33], which in turn affect their performance in discriminative tasks. The Dunning–Kruger effect also posits that as learning continues, individuals’ self-evaluations slowly rise and become more objective. In other words, the period given for people to learn the persuasion knowledge in this study may have been too short, resulting in a relatively limited improvement in misinformation discrimination. Researchers can consider increasing the intervention duration in the future in order to enhance the participants’ mastery of persuasion knowledge.

### 4.2. Underlying Mechanisms of the Effect of the Inoculation Intervention

The present study found that perceived threat did not mediate the effect of the inoculation intervention on misinformation discrimination, which is inconsistent with H2. Another study found similar results, as perceived threat induced by inoculation treatments was not significantly correlated with resistance, and this was despite successful resistance generation by the treatments [34].

One possible explanation is that this study used the traditional perceived threat scale developed by Compton and Ivanov [19], which may not have been suitable for the inoculation intervention proposed. Specifically, this traditional threat measurement focuses on concepts related to physical safety, not on motivations to defend one’s own attitudes, using words (e.g., scary and harmful) that may be more appropriate for responses to physical or fear-induced threats than attitudinal vulnerability [34]. O’Keefe [35] also argued that the presence of negative words in the items of this traditional perceived threat measurement (e.g., dangerous) triggers perceived threat, meaning that it cannot explain whether the elevated threat is caused by the items or by previous manipulations. In short, although researchers continue to extensively conduct research on inoculation [12,36], there is scope for improvement in the implementation and measurement of the concept of threat in this theory [19]. Scholars could further explore and validate these measurements of the threat concept in the near future.

The results of this study suggest that persuasion knowledge mediates the effect of the inoculation intervention on the ability to recognize misinformation, partially supporting Hypothesis (b). This also empirically validates previous claims in the literature that inoculation increases individual persuasion knowledge [37] and that individuals with high persuasion knowledge are more likely to accurately classify truthful and rumored information [22]. This finding contributes to the inoculation theory by providing theoretical support for the mechanisms underlying the effect of the inoculation process. Practical stakeholders can fully use theories on persuasion knowledge to help individuals improve their persuasion knowledge of misinformation, which may help to more effectively decrease the spread of misinformation. As expressed in a related study by the American Press Institute, improving the knowledge and understanding of information topics, generation strategies, and spread methods is key to helping the public discern misinformation [38].

### 4.3. Implications

Regarding theoretical implications, this study verifies the effectiveness of broad-spectrum inoculation-based interventions in confronting misinformation among the general public in the context of the COVID-19 pandemic. This serves as a powerful test of the applicability of the inoculation theory in the face of new challenges. The study also extends the inoculation theory with empirical evidence on whether the variables of perceived threat and persuasion knowledge play roles in the internal mechanisms of the effect of inoculation. Scholars have generally overlooked the topic of the underlying mechanisms of inoculation interventions and empirical examinations on this matter. This study establishes a link between the inoculation theory and the persuasion knowledge model, demonstrating the important role of persuasion knowledge on the effectiveness of inoculation-based interventions. This, in turn, provides a scientific basis for the development of more effective interventions.

In addition, this study introduces a new misinformation type, spreading false knowledge, into the traditional broad-spectrum inoculation intervention. On the one hand, this is a type of misinformation that is characteristic of the times in the context of the pandemic [4]. Unconfirmed information about the new coronavirus characteristics, modes of transmission, and methods of prevention and treatment were proliferating at the beginning of the outbreak, seriously affecting the effectiveness of personal protection. On the other hand, much of the health-related misinformation appears to be science education but, rather, is spread content that is misaligned with common scientific knowledge [39]. This study confirms the existence of much misinformation that spreads false knowledge by coding the misinformation related to the pandemic. The addition helps prevent individuals from blindly believing science-based articles on social media, subsequently helping to improve information literacy and protect personal health [40] in an environment of exploding misinformation.

Regarding practical implications, on the one hand, this study configures a meaningful attempt to provide stakeholders with efficient methodologies to respond to the infodemic associated with COVID-19. In this study, we summarized the characteristics of misinformation related to the COVID-19 pandemic and developed a self-help, online fake news game based on the broad-spectrum inoculation theory, along with verifying its effectiveness in enhancing misinformation discrimination. This may serve as useful data, from a psychological perspective, for stakeholders invested (e.g., government departments, media, and academia) in preventing, controlling, and resisting the influence of misinformation and the negative effects it may bring to society if it becomes widespread [4]. On the other hand, false health beliefs brought about by misinformation are an important factor influencing individual health behaviors [3], and the inoculation intervention developed in this study offers a convenient way to enhance personal protective behaviors during a pandemic. Especially with regard to vaccination, anti-scientific beliefs about vaccination are closely linked to the rampant spread of certain infectious diseases, such as measles [41]. Misinformation inoculation is particularly good at consolidating common sense perceptions, and a great deal of research has been conducted on the topic of vaccination [42]. Vaccination is now a central way to respond in advance to the spread of the COVID-19 virus, and online inoculation interventions are a very promising psychological way to improve perceptions of vaccination and provide new ideas for enhancing epidemic protection behaviors [43].

This research also validates the online game format as an effective approach for inoculation interventions, as described in prior research [15]. Internet-based guided intervention approaches have been gaining increasing attention in recent years and are considered promising because of the widespread availability of the Internet and its lack of geographical restrictions [44]. Specifically, we empirically verify the feasibility of game-based interventions; demonstrate that games are a relevant method to take inoculation interventions out of laboratory settings; and show that this format can be useful to deliver these interventions to the general public in a more fun, easy, and attractive way.

### 4.4. Limitations

Some limitations of this study must be acknowledged. First, perceived threat was measured using a traditional scale, as remarked above; some scholars have suggested [17,34] that the traditional threat measure should be modified to a motivated threat approach during the inoculation intervention (e.g., “*I want to protect my current attitudes from attacks*,” and “*I feel motivated to resist claims about XX*”) [34]. It is recommended that researchers analyze and compare both threat measures and results in future studies.

Second, scholars invested in studying the inoculation theory can further track the relationship between the intervention duration and effects. Academicians have demonstrated that both the duration and density of the intervention influence the intervention effect [45], and the current study found that Studies 1 (short priming) and 2 (five-day intervention) had different intervention effects. Therefore, future investigations can be related to finding the optimal duration and density of inoculation to obtain optimized intervention effects and determining whether “booster shots” are needed to prolong the intervention effect [46].

Third, scholars may also consider including people over 55 years of age, who are the most susceptible to misinformation and may benefit the most from inoculation interventions [15]. Prior research also remarked that older adults deserve attention in future rumor intervention studies, which could endeavor to develop research methods and interventions that are more appropriate for this group [47]. These remarks are consistent with the original intent of broad-spectrum inoculation to help more people master the skill of distinguishing misinformation, thus warranting attention from those interested in the topic.

Fourth, persuasive intent behavior is an important way to detect misinformation discernment, which is measured by the intent of reposting messages. However, reposting behavior possesses strong individual differences and is influenced by users’ personalities and user habits, as well as by contextual factors, such as message topics and browsing software [48]. Future research could consider selecting critical influences as covariates to control for or exploring other indicators that represent misinformation discernment.

Finally, the generalizability of the results is a limitation because the sample was exclusively Chinese people residing in China. It is unclear whether the results would be replicated in samples from other countries. Cross-cultural studies are needed to further investigate the intervention of misinformation and its mechanisms. Pandemic development and response policies vary from country to country [49], and the misinformation associated with it may derive different characteristics. Rumors have a deep historical and cultural background [50], and they need to be rooted in the appropriate culture in order to gain widespread trust and induce broad dissemination. Studies have found significant differences in social media disinformation between the United States, the United Kingdom, Germany, and Austria, even though these countries have Western democracies [51]. Therefore, the cross-cultural applicability of this study needs to be further explored in future research.

## 5. Conclusions

This study demonstrated the applicability of the inoculation theory to COVID-19-related misinformation with an online priming study and randomized control trial, with the latter applying a game-based intervention to provide practical experience in curbing the effects of an infodemic. This study also extends our understanding of the mechanisms underlying the effectiveness of the inoculation theory, depicting that the inoculation intervention enhances misinformation discrimination by improving persuasion knowledge at the individual level. Accordingly, it provides empirical grounding for the hypothesis on the effects of broad-spectrum inoculation interventions.

## Figures and Tables

**Table 1:** Descriptive statistics at different time points in Study 1.

	Control Group		Priming Group	
	*M*	*SD*	*M*	*SD*
ARM T1	4.670	0.629	4.629	0.626
ARM T2	4.640	0.570	4.937	0.625
ARM T3	4.627	0.672	4.904	0.602
PIM T1	4.461	0.534	4.423	0.544
PIM T2	4.458	0.515	4.607	0.577
PIM T3	4.348	0.498	4.488	0.564

Note: ARM means the ability to recognize misinformation; PIM means the persuasive intent of misinformation; T1 means pretest; T2 means posttest; T3 means one-week follow-up.

**Table 2:** Descriptive statistics at different time points in Study 2.

	Control Group		Intervention Group	
	*M*	*SD*	*M*	*SD*
ARM T1	4.606	0.623	4.610	0.560
ARM T2	4.549	0.541	4.829	0.713
ARM T3	4.577	0.692	4.766	0.783
PIM T1	4.501	0.640	4.563	0.570
PIM T2	4.441	0.536	4.752	0.709
PIM T3	4.373	0.579	4.616	0.705

## Data Availability

Requests for data and materials should be directed to the corresponding author or first author.
